# Nitrite Promotes ROS Production to Potentiate Cefoperazone-Sulbactam-Mediated Elimination to Lab-Evolved and Clinical-Evolved Pseudomonas aeruginosa

**DOI:** 10.1128/spectrum.02327-21

**Published:** 2022-07-05

**Authors:** Su-fang Kuang, Xia Li, Ding-Yun Feng, Wen-Bin Wu, Hui Li, Bo Peng, Xuan-xian Peng, Zhuang-gui Chen, Tian-tuo Zhang

**Affiliations:** a The Third Affiliated Hospital and State Key Laboratory of Bio-Control, School of Life Sciences, Southern Marine Science and Engineering Guangdong Laboratory (Zhuhai), Sun Yat-sen University, University City, Guangzhou, People’s Republic of China; b Laboratory for Marine Fisheries Science and Food Production Processes, Qingdao National Laboratory for Marine Science and Technology, Qingdao, China; Riverside University Health System, Medical Center – University of California

**Keywords:** *Pseudomonas aeruginosa*, antibiotic resistance, cefoperazone-sulbactam, electron transport chain, nitrite, reprogramming metabolomics, the pyruvate cycle

## Abstract

Cefoperazone-sulbactam (SCF)-resistant Pseudomonas aeruginosa poses a big challenge in the use of SCF to treat infection caused by the pathogen. We have recently shown exogenous nitrite-enabled killing of naturally and artificially evolved Pseudomonas aeruginosa strains (AP-R_CLIN-EVO_ and AP-R_LAB-EVO_, respectively) by SCF. However, the underlying mechanism is unknown. Here, reprogramming metabolomics was adopted to investigate how nitrite enhanced the SCF-mediated killing efficacy. Nitrite-reprogrammed metabolome displayed an activated pyruvate cycle (P cycle), which was confirmed by elevated activity of pyruvate dehydrogenase (PDH), α-ketoglutarate dehydrogenase, succinate dehydrogenase, and malate dehydrogenase. The activated P cycle provided NADH for the electron transport chain and thereby increased reactive oxygen species (ROS), which potentiated SCF to kill AP-R_CLIN-EVO_ and AP-R_LAB-EVO_. The nitrite-enabled killing of AP-R_CLIN-EVO_ and AP-R_LAB-EVO_ by SCF was inhibited by PDH inhibitor furfural and ROS scavenger N-Acetyl-L-cysteine but promoted by ROS promoter Fe^3+^. SCF alone could not induce ROS, but SCF-mediated killing efficacy was enhanced by ROS. In addition, the present study demonstrated that nitrite repressed antioxidants, which were partly responsible for the elevated ROS. These results reveal a nitrite-reprogrammed metabolome mechanism by which AP-R_CLIN-EVO_ and AP-R_LAB-EVO_ sensitivity to SCF is elevated.

**IMPORTANCE** Antibiotic-resistant Pseudomonas aeruginosa has become a real concern in hospital-acquired infections, especially in critically ill and immunocompromised patients. Understanding antibiotic resistance mechanisms and developing novel control measures are highly appreciated. We have recently shown that a reduced nitrite-dependent NO biosynthesis contributes to cefoperazone-sulbactam (SCF) resistance, which is reverted by exogenous nitrite, in both naturally and artificially evolved P. aeruginosa strains (AP-R_CLIN-EVO_ and AP-R_LAB-EVO_, respectively). However, the mechanism is unknown. The present study reports that the nitrite-enabled killing of AP-R_CLIN-EVO_ and AP-R_LAB-EVO_ by SCF is attributed to the promoted production of reactive oxygen species (ROS). Nitrite activates the pyruvate cycle to generate NADH for the electron transport chain, which in turn promotes ROS generation. Nitrite-potentiated SCF-mediated killing is decreased by pyruvate dehydrogenase inhibitor furfural and ROS scavenger N-Acetyl-L-cysteine but increased by ROS promoter Fe^3+^. Furthermore, SCF-mediated killing is promoted by H_2_O_2_ in a dose-dependent manner. In addition, the combination of nitrite and H_2_O_2_ greatly enhances SCF-mediated killing. These results not only disclose a nitrite-ROS-potentiated SCF-mediated killing, but also show SCF-mediated killing is dependent upon ROS.

## INTRODUCTION

Pseudomonas aeruginosa is an opportunistic pathogen that causes severe infections in humans, especially in cystic fibrosis patients. Antibiotic-resistant P. aeruginosa isolates are frequently isolated in clinic and environments since it rapidly develops multidrug resistance under antibiotic selective pressure (1, 2). These resistant strains are insensitive to most classes of antibiotics. However, no new antibiotics are available to combat the growing threat of the antibiotic resistance. Therefore, combating these multidrug-resistant P. aeruginosa is an urgent issue in clinical practice.

P. aeruginosa has the ability to adapt to its environment through a versatile energy metabolism network (3), suggesting that this bacterium may manipulate metabolism to cope with antibiotic stress. Han et al. used a metabolomics approach in combination with lipidomics or transcriptomics to investigate polymyxin-resistant P. aeruginosa. They showed that polymyxin treatment causes significant perturbations in the biosynthesis of lipids, lipopolysaccharide and peptidoglycan, central carbon metabolism, and oxidative stress. They proposed decreased phospholipid level as a consequence of polymyxin resistance (4, 5). Hussein et al. utilized untargeted metabolomics to investigate the mechanism(s) of synergy between polymyxin B and tamoxifen or sertraline against polymyxin-resistant multidrug-resistant (MDR) cystic fibrosis (CF) P. aeruginosa isolates. The primary mechanisms with tamoxifen involve disruption of cell envelope biogenesis and inhibition of lipopolysaccharide (LPS) modifications. In combination with sertraline, polymyxin B impairs glycerophospholipids and fatty acids and the pantothenate and coenzyme A pathways (6, 7). These studies may provide valuable information about metabolic pathways leading to an understanding of the adaptations of bacterial strains to antibiotic stress.

Recently developed reprogramming metabolomics can identify crucial biomarkers to revert or restore metabolomes against stresses (8–12), including antibiotic-resistant metabolomes reprogrammed to antibiotic-sensitive metabolomes to potentiate antibiotic-mediated killing efficacy (13–18). In a recent study, we have adopted an approach to investigate metabolic profiles in naturally and artificially evolved strains carrying cefoperazone-sulbactam (SCF) resistance (AP-R_CLIN-EVO_ and AP-R_LAB-EVO_, respectively) from the same parental strain (AP-R_CLIN_). AP-R_CLIN_ and AP-R_CLIN-EVO_ are isolated from lower respiratory secretions of the patient by using a bronchofiberscope at 3-day intervals. AP-R_LAB-EVO_ is obtained through sequential propagation of AP-R_CLIN_ in medium with SCF. Reduced NO is identified as an overlapped characteristic feature of the two evolved strains, which is attributed to nitrite-dependent NO biosynthesis instead of an arginine-dependent NO pathway. Exogenous nitrite promotes NO and thereby potentiates SCF-mediated killing (19). However, the mechanism underlying the nitrite-potentiated killing is unknown.

Here, gas chromatography-mass spectrometry (GC-MS) based metabolomics are used to compare metabolic profiles between nitrite-induced and control metabolomes to understand the metabolic differences. We demonstrate that nitrite-potentiated SCF-mediated killing is attributed to promotion of ROS and SCF is a ROS-dependent antibiotic.

## RESULTS

### Nitrite-induced metabolic profiles in AP-R_CLIN-EVO_ and AP-R_LAB-EVO_.

**FIG 1:**
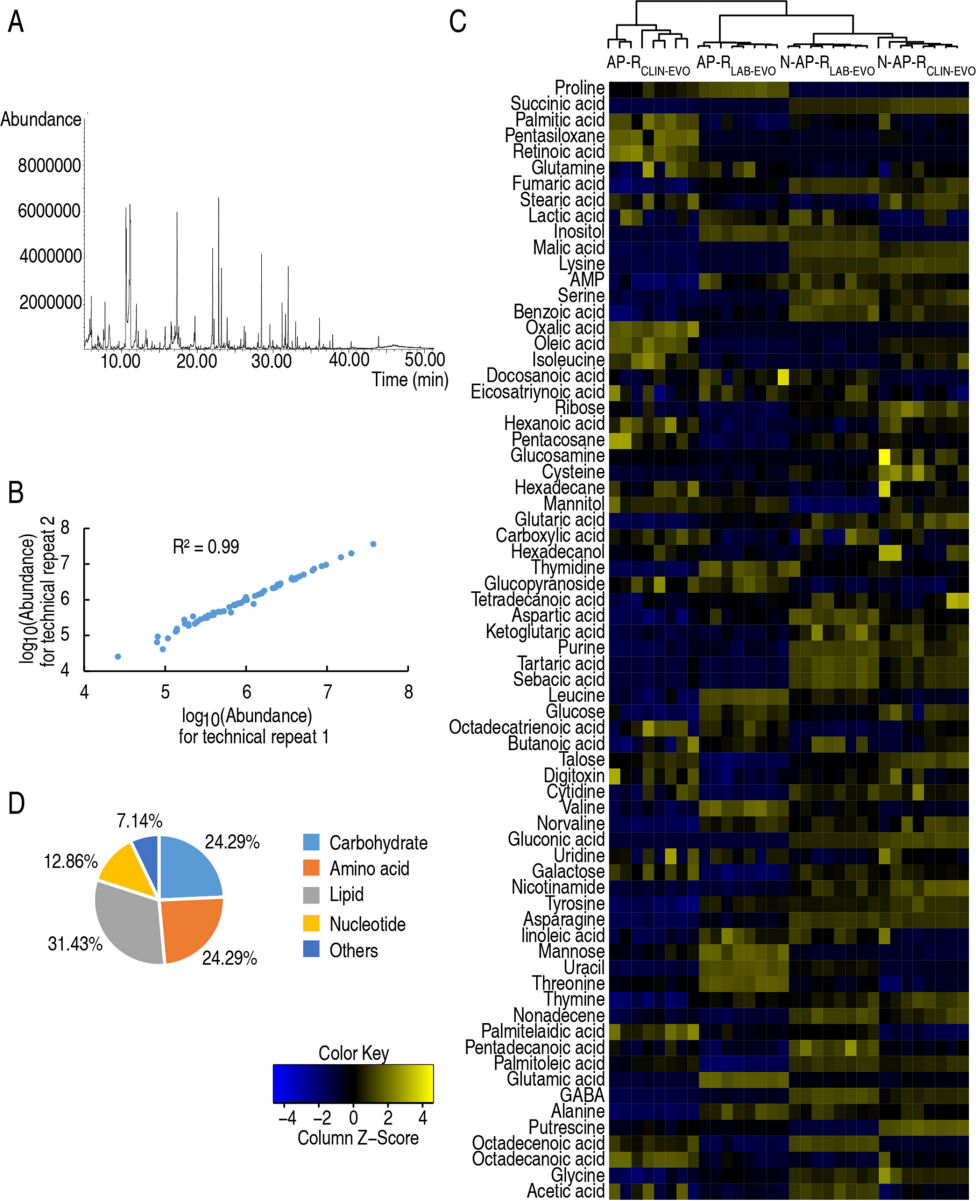
Metabolic profiles between nitrite-induced metabolomics in AP-R_CLIN-EVO_ and AP-R_LAB-EVO_. (A) Representative total ion current chromatogram. (B) Pearson correlation coefficient between technical replicates. (C) Heat map of unsupervised hierarchical clustering of all metabolites (row). Yellow and blue colors indicate increase and decrease of the metabolites scaled to mean and standard deviation of row metabolite level, respectively (see color scale). (D) Categories of all of the identified metabolites.

AP-R_CLIN-EVO_ and AP-R_LAB-EVO_ were cultured in medium with and without nitrite and designed as N-AP-R_CLIN-EVO_, N-AP-R_LAB-EVO_ and AP-R_CLIN-EVO_, AP-R_LAB-EVO,_ respectively. Then, GC-MS-based metabolomics were adopted to characterize nitrite-induced metabolic profiles in N-AP-R_CLIN-EVO_ and N-AP-R_LAB-EVO_ compared to those of AP-R_CLIN-EVO_ and AP-R_LAB-EVO_, respectively. Four biological samples with two technical replicates were performed for each group, yielding a total of 32 data sets. A total of 230 aligned individual peaks were achieved (Fig. 1A). The correlation coefficients between technical duplication varied between 0.95 and 0.99, indicating the reproducibility of the data (Fig. 1B). After removing the internal standard ribitol and any known artificial peaks, 70 metabolites were identified at each sample. N-AP-R_CLIN-EVO_ and N-AP-R_LAB-EVO_ were first clustered together and then with AP-R_LAB-EVO_ and last with AP-R_CLIN-EVO_ (Fig. 1C). As shown in the metabolite classification chart, the proportions of carbohydrate, amino acid, lipid, nucleotide, and other metabolites were 24.29%, 24.29%, 31.43%, 12.86%, and 7.14%, respectively (Fig. 1D). These results indicate that nitrite induces similar metabolic shift between N-AP-R_CLIN-EVO_ and N-AP-R_LAB-EVO_, which is different from AP-R_CLIN-EVO_ and AP-R_LAB-EVO_.

### Nitrite-induced differential metabolomes in N-AP-R_CLIN-EVO_ and N-AP-R_LAB-EVO_.

**FIG 2:**
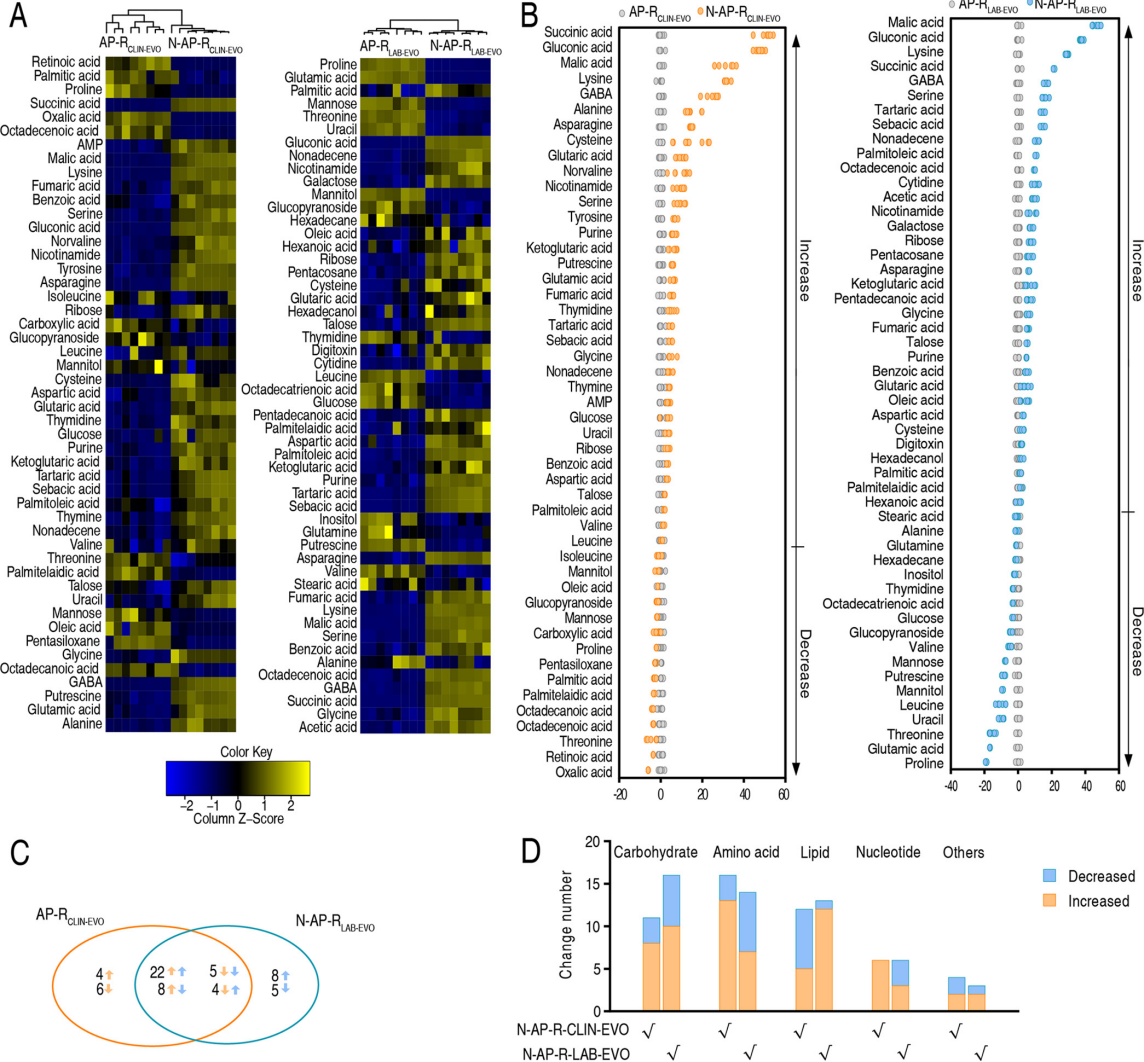
Nitrite-induced differential metabolomics in AP-R_CLIN-EVO_ and AP-R_LAB-EVO._ (A) Heat map showing differential metabolites. The metabolites are organized top to bottom according to hierarchical clustering. Yellow and blue colors indicate increase and decrease of metabolites relative to the median metabolite level, respectively (see color scale). (B) Z-score plot of differential metabolites based on control. The data of AP-R_CLIN-EVO_ (left) and AP-R_LAB-EVO_ (right) groups are separately scaled to the mean and standard deviation of control. Each point represents one metabolite in one technical repeat and colored by sample types. (C) Venn diagram for comparison of nitrite-induced differential metabolites between N-AP-R_CLIN-EVO_ (yellow color) and N-AP-R_LAB-EVO_ (blue color). (D) Number of differential abundance of metabolites.

A Kruskal–Wallis test (*P < *0.05) was used to identify metabolites of differential abundance in N-AP-R_CLIN-EVO_ and N-AP-R_LAB-EVO_. Compared with AP-R_CLIN-EVO_ and AP-R_LAB-EVO_, 49 and 52 differential metabolites were detected in N-AP-R_CLIN-EVO_ and N-AP-R_LAB-EVO_, respectively (Fig. 2A). To measure the number of standard deviations between a value and the mean, Z-score calculation was used. It varied between −6.02 and 51.12 in N-AP-R_CLIN-EVO_ and −18.96 and 46.09 in the N-AP-R_LAB-EVO_ group. The top four increased metabolites were succinic acid, gluconic acid, malic acid, and lysine in N-AP-R_CLIN-EVO_ and malic acid, gluconic acid, lysine, and succinic acid in N-AP-R_LAB-EVO_ (Fig. 2B), where succinic acid and malic acid work for the pyruvate cycle (the P cycle), a recently defined cycle in providing respiratory energy in bacteria (20). Among these differential metabolites, 39 overlapped between the two strains, and 10 and 13 were specific to N-AP-R_CLIN-EVO_ and N-AP-R_LAB-EVO_, respectively. Among the 39 overlapping metabolites, 22 were increased and 5 were decreased in both strains, while 8 were upregulated and 4 were downregulated in N-AP-R_CLIN-EVO_ but oppositely changed in N-AP-R_LAB-EVO_ (Fig. 2C). Number and distribution of these differential abundances of metabolites are shown in Fig. 2D. These results indicate that nitrite induces metabolic shift, where elevated succinic acid and malic acid in the P cycle are a characteristic feature in N-AP-R_CLIN-EVO_ and N-AP-R_LAB-EVO_.

### Nitrite-induced pathways in N-AP-R_CLIN-EVO_ and N-AP-R_LAB-EVO_.

**FIG 3:**
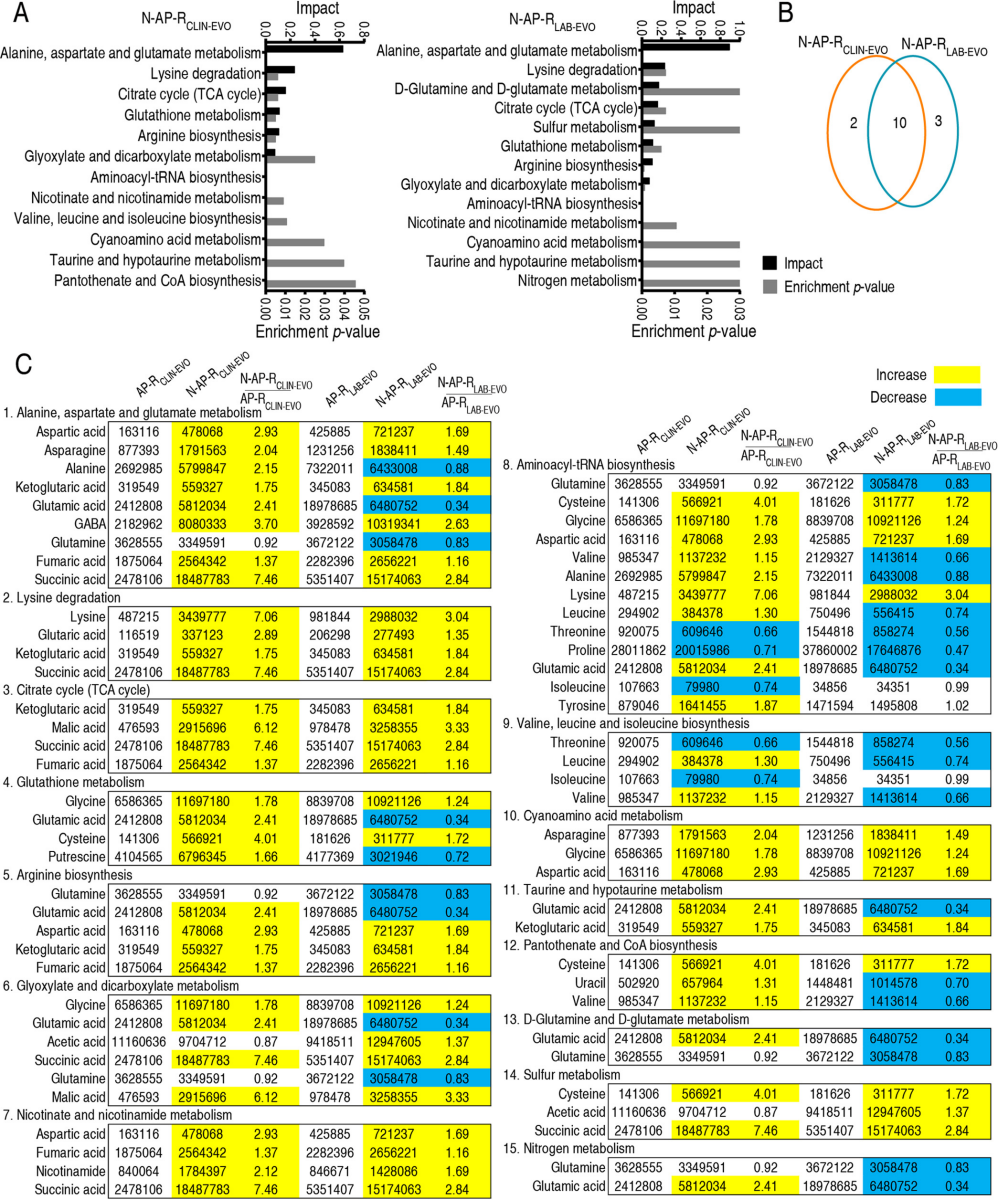
Overlapped enriched pathways between N-AP-R_CLIN-EVO_ and N-AP-R_LAB-EVO_. (A) Pathway enrichment of differential metabolites between N-AP-R_CLIN-EVO_ and N-AP-R_LAB-EVO_. (B) Venn diagram for comparison of nitrite-induced pathways between N-AP-R_CLIN-EVO_ (yellow color) and N-AP-R_LAB-EVO_ (blue color). (C) Integrative analysis of metabolites in significantly enriched pathways. Yellow and blue colors indicate increased and decreased metabolites (*P* value <0.05), respectively.

Pathway enrichment analysis is crucial to cluster the changes in metabolic pathways. Twelve and 13 metabolic pathways were enriched in N-AP-R_CLIN-EVO_ and N-AP-R_LAB-EVO_, respectively, whereas 10 pathways (alanine, aspartate, and glutamate metabolism; lysine degradation; tricarboxylic acid acid [TCA] cycle; glutathione metabolism; arginine biosynthesis; glyoxylate and dicarboxylate metabolism; aminoacyl-tRNA biosynthesis; nicotinate and nicotinamide metabolism; cyanoamino acid metabolism; and taurine and hypotaurine metabolism) overlapped. Besides, valine, leucine, and isoleucine biosynthesis and pantothenate and CoA biosynthesis were enriched in N-AP-R_CLIN-EVO_, while d-glutamine and d-glutamate metabolism, sulfur metabolism, and nitrogen metabolism were enriched in N-AP-R_LAB-EVO_ (Fig. 3A and B). Metabolites in these enriched metabolic pathways are listed in Fig. 3C. Among them, all metabolites of lysine degradation, the TCA cycle, nicotinate and nicotinamide metabolism, and cyanoamino acid metabolism were increased in N-AP-R_CLIN-EVO_ and N-AP-R_LAB-EVO_. Notably, half of the metabolites in lysine degradation and nicotinate and nicotinamide metabolism belong to the TCA cycle (Fig. 3C). Here, we would like to mainly investigate the TCA cycle. Since the P cycle covers the TCA cycle in providing respiratory energy in bacteria (20), the P cycle is used as a key clue for the following study.

### Nitrite-induced biomarkers in N-AP-R_CLIN-EVO_ and N-AP-R_LAB-EVO_.

**FIG 4:**
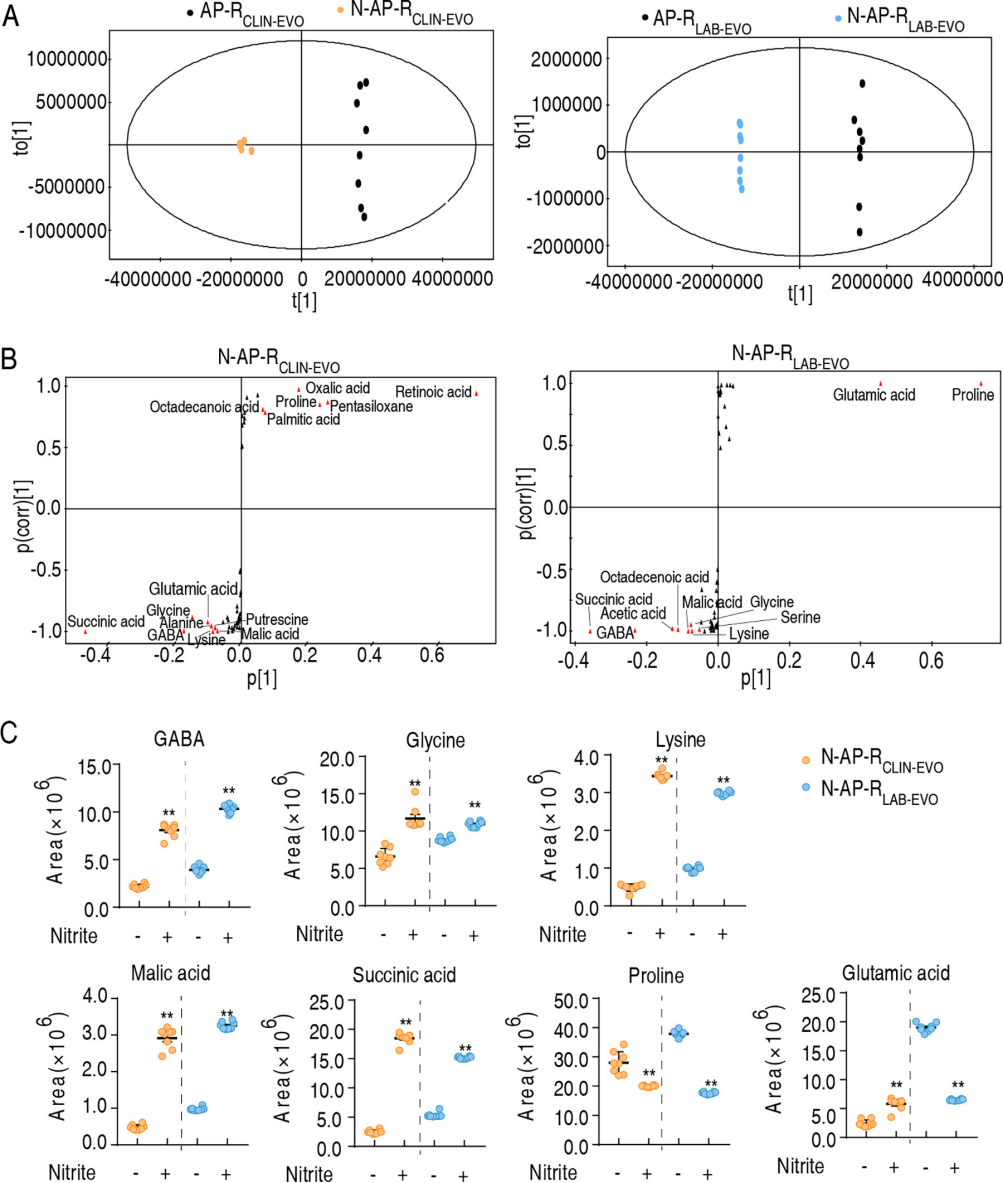
Overlapped biomarkers between N-AP-R_CLIN-EVO_ and N-AP-_RLAB-EVO_. (A) OPLS-DA of nitrite-induced metabolomes in AP-R_CLIN-EVO_ (left) and AP-_RLAB-EVO_ (right). Each dot represents the biological and technical replicate analysis of samples in the plot. (B) S-plot generated from OPLS-DA. Predictive component p [1] and correlation p(corr) [1] differentiate the nitrite-induced group from the control group in AP-R_CLIN-EVO_ (left) and AP-R_LAB-EVO_ (right). Triangles represents individual metabolites, where potential biomarkers are highlighted in red, which are greater or equal to 0.05 and 0.5 for absolute value of covariance p and correlation p(corr), respectively. Otherwise, triangles are marked in black. (C) The scatterplot of overlapped biomarkers of nitrite-induced metabolomes between N-AP-R_CLIN-EVO_ and N-AP-R_LAB-EVO_. Results are displayed as mean ± SEM of four biological replicas and two technical replicates, and significant differences are identified: *, *P* <0.05; **, *P < *0.01.

Pattern recognition method is a useful tool to identify biomarkers in metabolomics analysis. Thus, orthogonal partial least square discriminant analysis (OPLS-DA) was adopted to recognize the sample patterns of metabolomes. Component t [1] separated N-AP-R_CLIN-EVO_ and N-AP-R_LAB-EVO_ from AP-R_CLIN-EVO_ and AP-R_LAB-EVO_ (Fig. 4A). Furthermore, discriminating variables were demonstrated by S-plot. In the plots of predictive correlation between p [1] and p(corr) [1], the red triangle indicates the differential metabolites that had larger weights (<–0.05 or >0.05) and higher relevance (<–0.5 or >0.5). Fourteen metabolites (alanine, GABA, glutamic acid, glycine, lysine, malic acid, putrescine, succinic acid, octadecanoic acid, oxalic acid, palmitic acid, pentasiloxane, proline, retinoic acid) and 10 metabolites (acetic acid, GABA, glycine, lysine, malic acid, octadecenoic acid, serine, succinic acid, glutamic acid, proline) were identified as biomarkers in N-AP-R_CLIN-EVO_ and N-AP-R_LAB-EVO_, respectively (Fig. 4B). Among them, seven biomarkers overlapped between N-AP-R_CLIN-EVO_ and N-AP-R_LAB-EVO_, and their abundance is shown as scatterplots in Fig. 4C. The presence of malic acid and succinic acid supporting the P cycle may be a key clue to its role in the resistance.

### Nitrite-induced metabolic flux in N-AP-R_CLIN-EVO_ and N-AP-R_LAB-EVO_.

**FIG 5:**
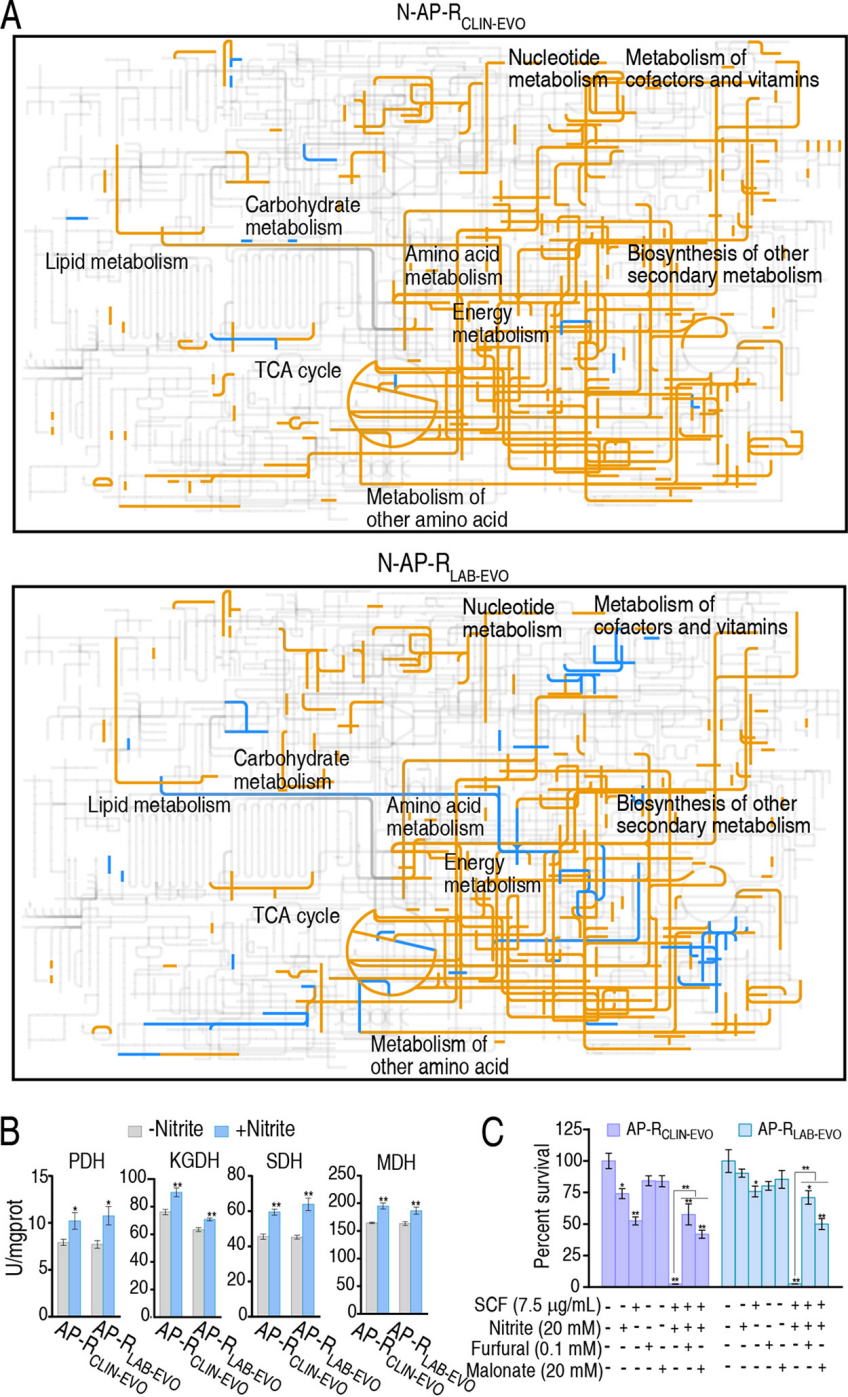
iPath analysis and enzyme activity determination. (A) iPath analysis showing comparison of nitrite-induced metabolomes between N-AP-R_CLIN-EVO_ and N-AP-R_LAB-EVO_. The yellow and blue lines mean upregulation and downregulation of metabolic pathways, respectively. (B) Activity of enzymes of PDH, KGDH, SDH, and MDH in the P cycle with or without nitrite (20 mM). (C) Percent survival of AP-R_CLIN-EVO_ and AP-R_LAB-EVO_ in the presence or absence of furfural or malonate plus SCF and nitrite. Result (C) is obtained using antibiotic bactericidal assay. Results are displayed as mean ± SEM of three biological replicas and significant differences are identified (*, *P < *0.05; **, *P < *0.01) as determined by two-tailed Student's *t* test (B) and analysis of variance (ANOVA) (C).

An online interactive tool, iPath3.0, was used to understand the nitrite-induced global metabolic flux of N-AP-R_CLIN-EVO_ and N-AP-R_LAB-EVO_. Exogenous nitrite activated almost all metabolic pathways, where more activated metabolism was detected in AP-R_CLIN-EVO_ than in AP-R_LAB-EVO_ (Fig. 5A). We further measured activity of pyruvate dehydrogenase (PDH), α-ketoglutarate dehydrogenase (KGDH), succinate dehydrogenase (SDH), and malate dehydrogenase (MDH) in the P cycle to validate the activation of the P cycle. Increased activity of the four enzymes was obtained in N-AP-R_CLIN-EVO_ and N-AP-R_LAB-EVO_ compared with AP-R_CLIN-EVO_ and AP-R_LAB-EVO_, respectively (Fig. 5B). Finally, furfural and malonate, which inhibit the activity of PDH and SDH, respectively, were used to block the P cycle. The inhibition elevated the viability of AP-R_LAB-EVO_ and AP-R_CLIN-EVO_ (Fig. 5C). These results validate the activated P cycle as the most characteristic consequence of the nitrite-induced metabolome.

### Activation of electron transport chain contributes to nitrite-potentiated SCF killing.

**FIG 6:**
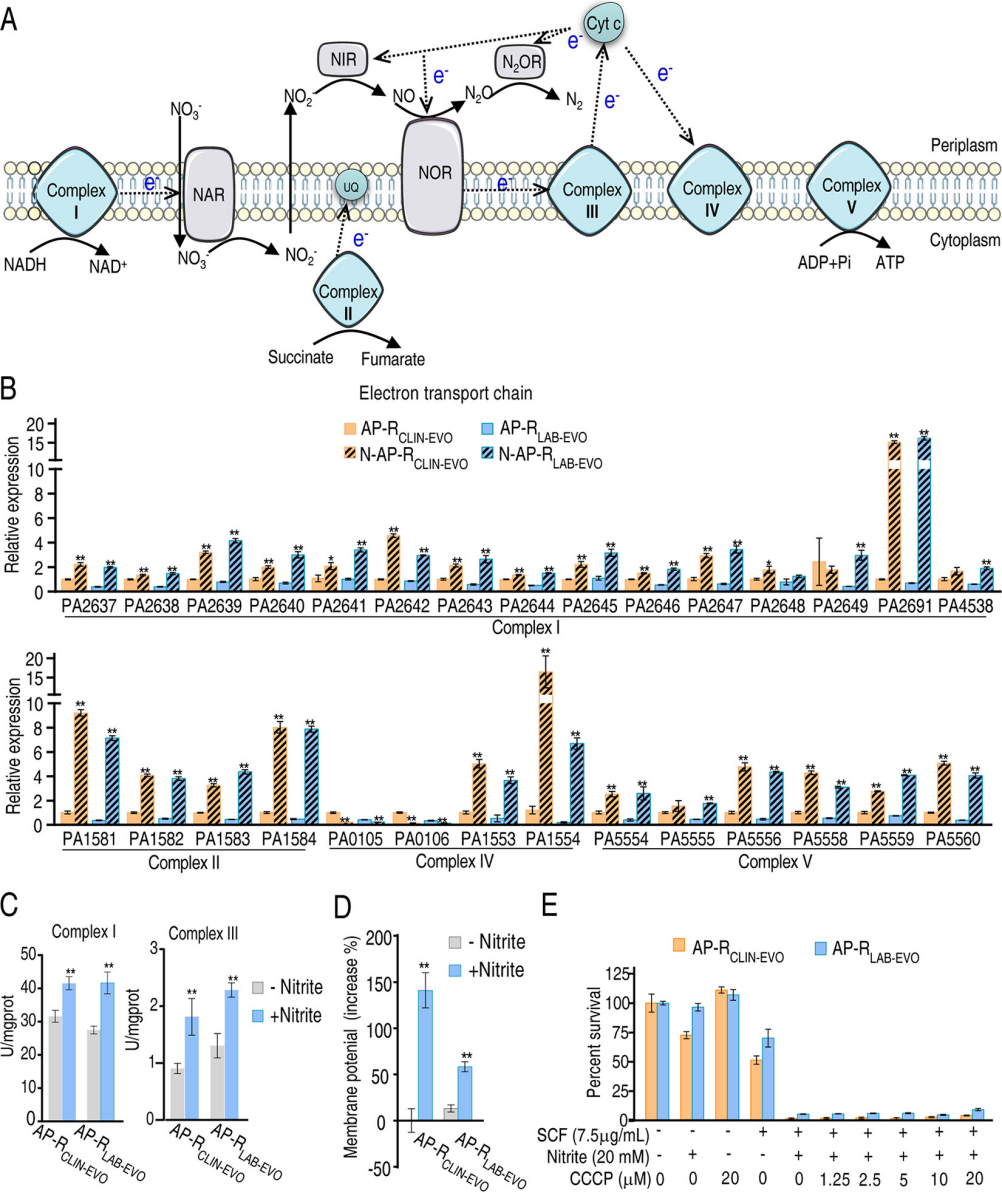
Effect of nitrite on the electron transport chain. (A) Diagram showing electron transport chain. (B) qRT-PCR for expression of genes encoding electron transport chain in the presence of nitrite. (C) Activity of complex II and complex III in electron transport chain in the presence of nitrite (20 mM). (D) Membrane potential in the presence of nitrite. (E) Percent survival of AP-R_CLIN-EVO_ and AP-R_LAB-EVO_ in the presence or absence of the indicated doses of CCCP plus nitrite (20 mM) and SCF (7.5 μg/mL). Result (E) is obtained using antibiotic bactericidal assay. Results are displayed as mean ± SEM of three (C–E) or four (B) biological replicas, and significant differences are identified (*, *P < *0.05; **, *P < *0.01) as determined by two-tailed Student's *t* test.

Nitrite does not activate the electron transport chain directly (Fig. 6A). Our recent report has indicated that NADH is lower in AP-R_CLIN-EVO_ and AP-R_LAB-EVO_ than their parent strain AP-R_CLIN_, whereas exogenous nitrite promotes higher NADH in AP-R_CLIN-EVO_ and AP-R_LAB-EVO_ than AP-R_CLIN_. Furthermore, nitrite induces expression of Cyt bc1 complex genes (19). Therefore, the activated P cycle should promote electron transport chain. To further demonstrate this, qRT-PCR was used to measure expression of electron transport chain genes in the presence of nitrite. Exogenous nitrite promoted the expression of most genes in N-AP-R_CLIN-EVO_ and N-AP-R_LAB-EVO_. Specifically, expression of all genes was upregulated except for PA2648 (unchanged in AP-R_LAB-EVO_) and PA2649 (unchanged in AP-R_CLIN-EVO_) of complex I, and PA0105 and PA0106 (lower in both strains) of complex IV (Fig. 6B). Furthermore, activity of complex 1 and complex III, which generate ROS, was measured. Higher activity of the two enzymes was detected in N-AP-R_CLIN-EVO_ and N-AP-R_LAB-EVO_ than in AP-R_CLIN-EVO_ and AP-R_LAB-EVO_ (Fig. 6C). Exogenous nitrite increased membrane potential (Fig. 6D). However, viability of AP-R_CLIN-EVO_ and AP-R_LAB-EVO_ was similar between the presence and absence of increasing doses of carbonyl cyanide m-chlorophenylhydrazone (CCCP) (Fig. 6E). These results indicate that nitrite-potentiated SCF-mediated killing is independent of membrane potential.

### Activated electron transport chain promotes ROS.

**FIG 7:**
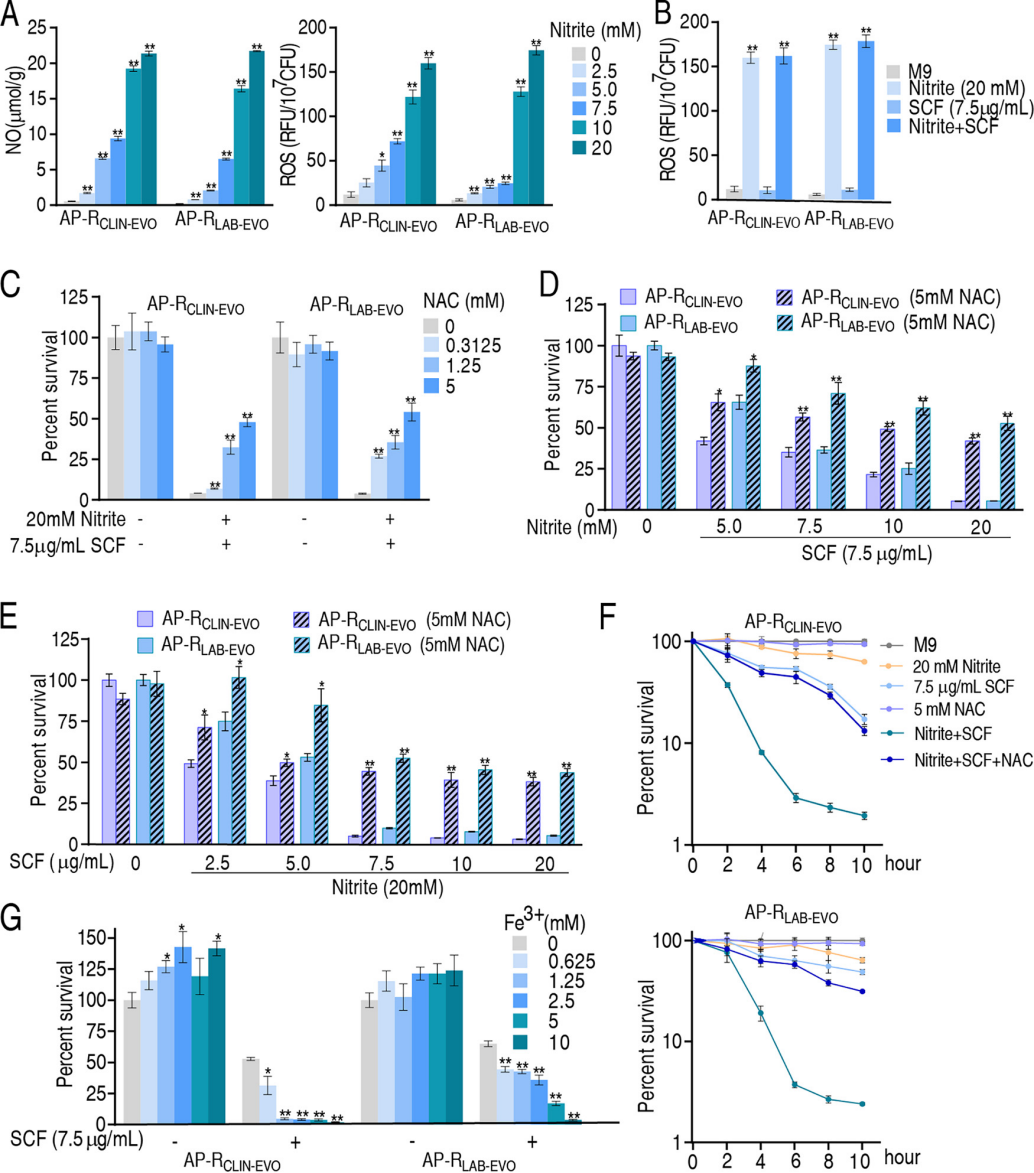
Role of ROS in the synergistic killing of SCF with nitrite. (A) NO and ROS of AP-R_CLIN-EVO_ and AP-R_LAB-EVO_ in the presence of the indicated doses of nitrite. (B) ROS of AP-R_CLIN-EVO_ and AP-R_LAB-EVO_ in the presence of SCF (7.5 μg/mL), nitrite (20 mM), and both. (C) Percent survival of AP-R_CLIN-EVO_ and AP-R_LAB-EVO_ in the indicated concentrations of NAC plus nitrite (20 mM) and SCF (7.5 μg/mL). (D) Percent survival of AP-R_CLIN-EVO_ and AP-R_LAB-EVO_ in the presence or absence of NAC (5 mM) and the indicated concentrations of nitrite plus 7.5 μg/mL SCF. (E) Percent survival of AP-R_CLIN-EVO_ and AP-R_LAB-EVO_ in the presence of NAC (5 mM), nitrite (20 mM), or both plus the indicated concentrations of SCF. (F) Percent survival of AP-R_CLIN-EVO_ and AP-R_LAB-EVO_ in the presence or absence of SCF (7.5 μg/mL), NAC (5 mM), and nitrite (20 mM) at the indicated time point. (G) Percent survival of AP-R_CLIN-EVO_ and AP-R_LAB-EVO_ in the presence or absence of SCF (7.5 μg/mL) and the indicated concentration of Fe^3+^ (C_6_H_10_FeNO_8_). Results (C–G) are obtained using antibiotic bactericidal assay. Results are displayed as mean ± SEM of three biological replicas, and significant differences are identified (*, *P < *0.05; **, *P < *0.01) as determined by analysis of variance (ANOVA).

It is possible that activation of the electron transport chain elevates ROS. As expected, nitrite promoted not only NO but also ROS of N-AP-R_CLIN-EVO_ and N-AP-R_LAB-EVO_ in a dose-dependent manner. Notably, NO level was parallel to ROS level (Fig. 7A). However, SCF alone did not increase ROS level and synergize nitrite to increase ROS (Fig. 7B), suggesting that only nitrite is responsible for the elevated ROS. ROS scavenger N-Acetyl-L-cysteine (NAC) rescued the nitrite-potentiated SCF-mediated killing to AP-RCLIN-EVO and AP-RLAB-EVO in a dose-dependent manner (Fig. 7C). Bacterial viability caused by the rescue was reduced with the increasing doses of nitrite or SCF (Fig. 7D and E). The NAC-mediated rescue was enhanced with the incubation time (Fig. 7F). However, Fe^3+^ as a ROS generator promoted SCF-mediated killing to AP-R_CLIN-EVO_ and AP-R_LAB-EVO_ in a dose-dependent manner in addition to its role in increasing growth rates (Fig. 7G). These results indicate that ROS plays a key role in the SCF-mediated killing efficacy.

### Nitrite inhibits antioxidants defense system.

**FIG 8:**
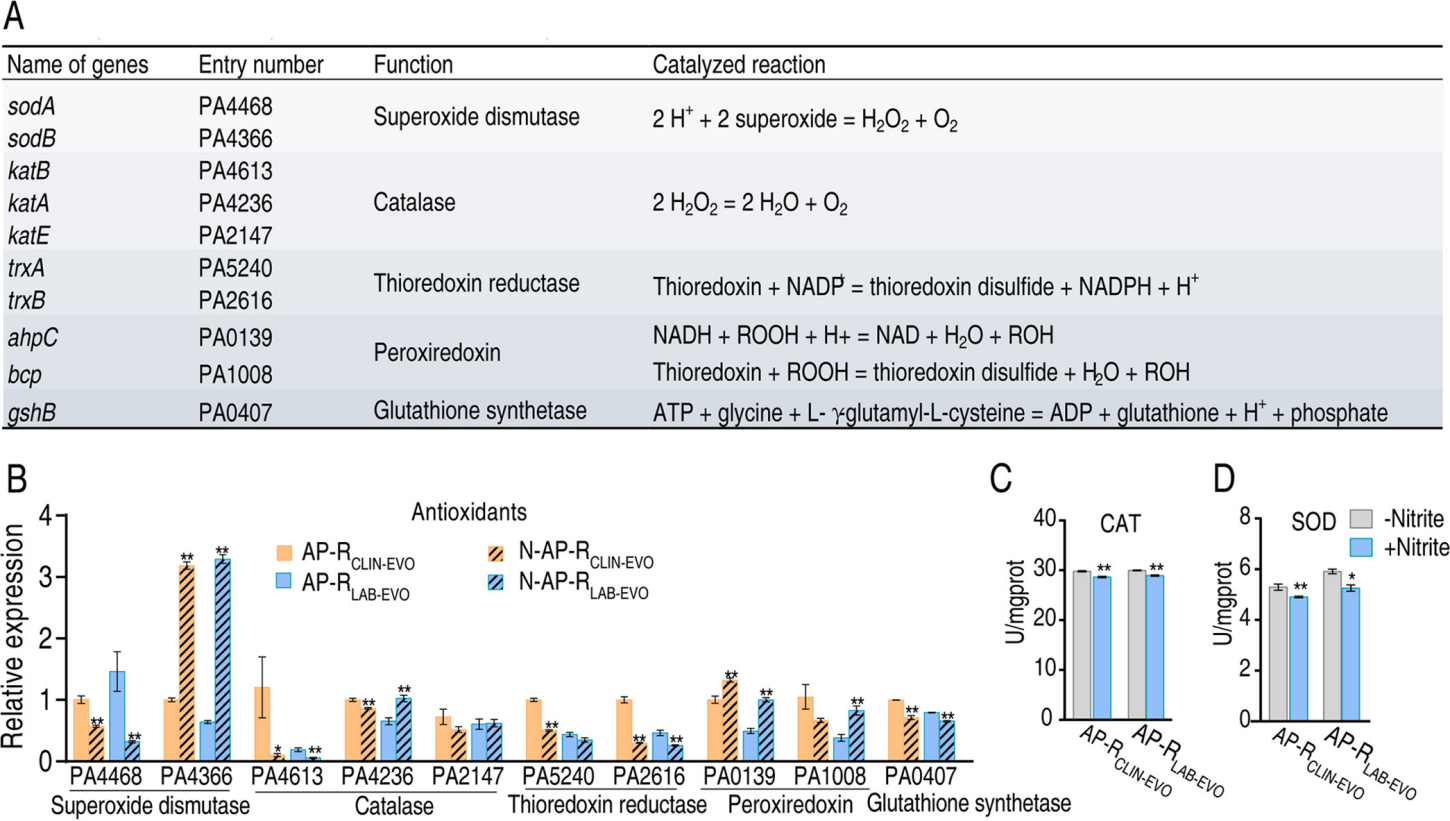
Role of antioxidant in the synergistic killing of SCF with nitrite. (A) Genes with antioxidant functions present in P. aeruginosa and the catalyzed reactions of the encoded enzymes. (B) Expression of genes encoding major classes of antioxidants in the presence of nitrite (20 mM). (C and D) Catalase (CAT) activity (C) and SOD activity (D) in the presence of exogenous nitrite (20 mM). Results are displayed as mean ± SEM of three (C and D) or four (B) biological replicas, and significant differences are identified (*, *P < *0.05; **, *P < *0.01) as determined by two-tailed Student's *t* test.

An antioxidant is an enzyme or cofactor that participates in eliminating ROS. To investigate whether nitrite-induced ROS production was linked to repression of antioxidants, expression of genes encoding antioxidants and activity of their enzymes were measured (Fig. 8A). Among 10 genes encoding five major classes of antioxidants, the expression of 2 genes encoding superoxide dismutase (SOD) was either reduced (PA4468) or increased (PA4366); the expression of three genes encoding catalase (CAT) was decreased (PA4613) and unchanged (PA2147), and reversal change (PA4236); the expression of two genes encoding thioredoxin reductase was decreased (PA5240 and PA2616); the expression of two genes encoding peroxiredoxin was increased (PA0139 and PA1008 only for AP-R_LAB-EVO_); and the expression of one gene encoding glutathione synthetase was decreased (PA0407) in the presence of nitrite (Fig. 8B). Meanwhile, the activity of SOD and CAT was lower in a medium with than without nitrite (Fig. 8C and D). These results indicate that nitrite inhibits degradation of ROS.

### H_2_O_2_ promotes SCF-mediated killing.

**FIG 9:**
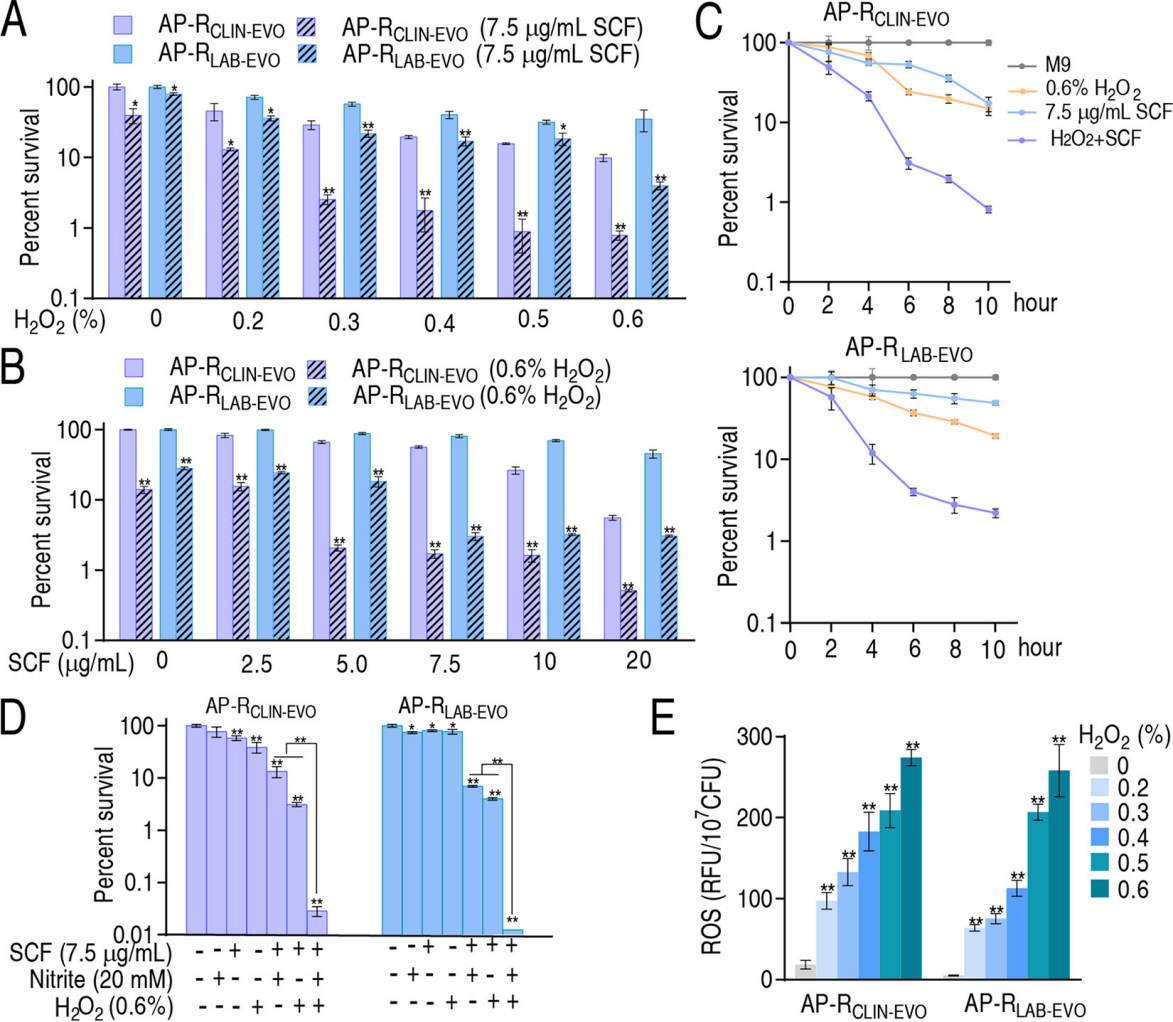
Role of ROS in SCF-mediated killing. (A) Percent survival of AP-R_CLIN-EVO_ and AP-R_LAB-EVO_ in the presence of SCF (7.5 μg/mL) and the indicated concentrations of H_2_O_2_. (B) Percent survival of AP-R_CLIN-EVO_ and AP-R_LAB-EVO_ in the presence of H_2_O_2_ (0.6%) and the indicated concentrations of SCF. (C) Percent survival of AP-R_CLIN-EVO_ and AP-R_LAB-EVO_ in the presence of SCF (7.5 μg/mL) and H_2_O_2_ (0.6%) at the indicated times. (D) Percent survival of AP-R_CLIN-EVO_ and AP-R_LAB-EVO_ in the presence of H_2_O_2_ (0.6%), nitrite (20 mM), and SCF (7.5 μg/mL). (E) ROS of AP-R_CLIN-EVO_ and AP-R_LAB-EVO_ in the presence of the indicated H_2_O_2._ Results (A–D) were obtained using antibiotic bactericidal assay. Results are displayed as mean ± SEM of three biological replicas, and significant differences are identified (*, *P < *0.05; **, *P < *0.01) as determined by analysis of variance (ANOVA).

The above results indicate that nitrite enhances ROS level, which potentiates SCF-mediated killing. Thus, it was necessary to confirm the ROS-potentiated SCF killing. To demonstrate this, H_2_O_2_ was used. Percent survival of AP-R_CLIN-EVO_ and AP-R_LAB-EVO_ was reduced with increasing H_2_O_2_ concentration when SCF was fixed at 7.5 μg/mL (Fig. 9A). The percent survival was also decreased at 0.6% H_2_O_2_ in a SCF dose-dependent manner (Fig. 9B). The killing efficacy was increased with the incubation time (Fig. 9C), and the combination of H_2_O_2_ with the ROS promoted by nitrite (Fig. 9D). The efficacy in 0.6% H_2_O_2_ was higher than that potentiated by 20 mM nitrite because 0.6% H_2_O_2_ caused higher ROS than 20 mM nitrite (Fig. 7B and 9E). These results support the conclusion that ROS contributes to SCF-mediated killing.

## DISCUSSION

Reprogramming metabolomics is a recently developed approach to promote antibiotic killing efficacy and restore host anti-infective ability through modulation of metabolic states (8, 9, 21–23). The present study uses this approach to understand mechanisms by which nitrite potentiates SCF-mediated killing to AP-R_CLIN-EVO_ and AP-R_LAB-EVO_. It was found that the P cycle and electron transport chain are activated, and thereby ROS is used as an effector to understand the nitrite-potentiated mechanism. Further evidence shows that the elevated ROS contributes to SCF-mediated killing. The finding that nitrite promotes the P cycle and electron transport chain and then increases ROS production to potentiate SCF-mediated killing was previously unknown.

The core finding of the present study is that nitrite activates the P cycle and electron transport chain. It is known that electron transport to nitrite and nitrous oxide involves c-type cytochromes that are to transfer electrons between complex III and complex IV of the respiratory chain (24). Replacement of oxygen by nitrite as final electron acceptor is determined under anaerobic conditions (25). However, whether nitrite activates the P cycle and electron transport chain is unknown except that the Cyt bc1 complex is activated by nitrite (19). Here, the activation of the P cycle is a characteristic feature in nitrite-reprogrammed metabolome, which is further demonstrated by the elevated activity of PDH, KGDH, SDH, and MDH in the P cycle in the presence of nitrite. These findings indicate that exogenous nitrite promotes the P cycle. The activated P cycle provides NADH for the electron transport chain. Further experiments on the expression of genes and the activity of enzymes show that the electron transport chain is activated. The activated electron transport chain elevates membrane potential, but the elevation is not related to SCF-mediated killing. Meanwhile, the activated electron transport chain produces more ROS, which contributes to the killing efficacy. These results also explain our recent finding that the Cyt bc1 complex of the electron transport chain is activated by nitrite (19). Therefore, nitrite activates the electron transport chain through the promotion of the P cycle.

The electron transport chain contributes to ROS production (26). ROS is related to antibiotic sensitivity in various species of bacteria (27–29). For P. aeruginosa, oxidative stress increases the development of antibiotic resistance (30, 31), but that ROS potentiates antibiotic-mediated killing efficacy is largely unknown. Kim et al. showed that PA01 is more resistant than PA14 to H_2_O_2_ as well as to polymyxin B (32). Hayakawa et al. utilized the stimulation by ROS from exposure to 1 mM H_2_O_2_ and promoted the acquisition of multidrug resistance in 20% of clinical isolates of P. aeruginosa. An anti-ROS agent, sodium zinc histidine dithiooctanamide, completely inhibits this acquisition of resistance (30). Liao et al., demonstrated that the mechanism of silver nanoparticles fighting against multidrug-resistant P. aeruginosa involves the disequilibrium of oxidation and antioxidation processes and the failure to eliminate the excessive ROS (33). However, polymyxin is not a clinically conventionally used antibiotic, and silver nanoparticles are an antibiotic candidate. The present study indicates that ROS potentiates SCF-mediated killing efficacy to AP-R_CLIN-EVO_ and AP-R_LAB-EVO_. SCF has a better effect against P. aeruginosa, *Enterobacteriaceae*, and Acinetobacter baumannii than cefoperazone alone and is widely used in clinics (34). Therefore, the elevation of ROS may be a potential approach to potentiate SCF against P. aeruginosa. However, AP-R_CLIN-EVO_ and AP-R_LAB-EVO_ originate from the same parent strain AP-R_CLIN_. Whether the mechanism is common to other clinically isolated multidrug-resistant P. aeruginosa strains awaits further investigation on more samples. Notably, ROS level is maintained by both biosynthesis and degradation. To explore whether nitrite breaks this balance, the present study quantifies the expression of genes and the activity of enzymes of the biosynthetic and degradation pathways. Nitrite promotes the biosynthesis of ROS but impairs the degradation pathway. Of note, the biosynthesis of ROS increases more than the degradation. These results suggest that nitrite promoting ROS generation is more crucial for the killing.

Interestingly, ROS is produced by immune cells during bacterial infection (35), which may pose oxidative stress to P. aeruginosa during invasion and thereby can be a possible source to facilitate the SCF-mediated killing. Actually, metabolites in alleviating or promoting ROS have been reported from lower vertebrates to Homo sapiens (12, 36, 37). Therefore, identifying metabolites promoting both anti-infective immune response and ROS generation can be a promising approach to enhance the killing by SCF, in addition to enhancing immune defense ability during infection, particularly during chronic infection.

**FIG 10:**
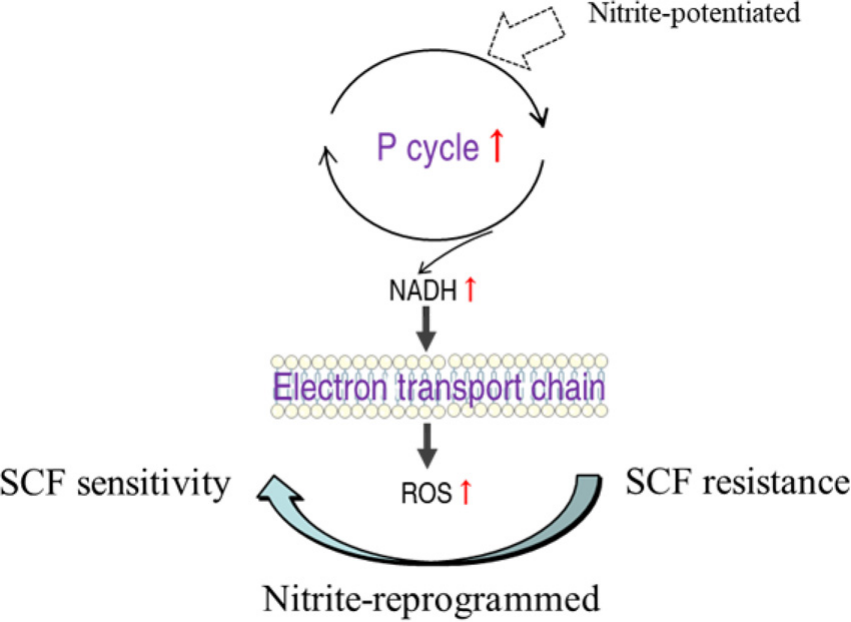
Diagram for the synergistic use of SCF and nitrite to kill AP-R_CLIN-EVO_ and AP-R_LAB-EVO_.

In summary, the present study reveals that exogenous nitrite activates the P cycle and electron transport chain, which elevates ROS production. ROS potentiates SCF to kill AP-R_CLIN-EVO_ and AP-R_LAB-EVO_ (Fig. 10). These findings not only identify a previously unknown metabolic regulation that nitrite modulates the P cycle and electron transport chain to promote ROS production, but also show that SCF is a ROS-dependent antibiotic.

## MATERIALS AND METHODS

### Source and culture conditions of bacterial strains.

P. aeruginosa strains AP-R_CLIN-EVO_ and AP-R_LAB-EVO_ were the same bacteria as the previous report (19) and were kept in our laboratory. These strains were cultured in fresh LB media at 37°C, with 200 rpm shaking until OD_600_ nm of 0.25–0.3. The cultures were reinoculated at 1:1,000 dilutions into 250 mL flasks and incubated for 16 h. The overnight bacteria were collected, washed three times with saline, and suspended in M9 medium (7 g/L K_2_HPO_4_, 3 g/L KH_2_HPO_4_, 1 g/L (NH_4_)_2_SO_4_, 0.1 g/L MgSO_4_, 0.588 g/L sodium citrate) to 0.2 of OD_600_ nm when metabolites or/and antibiotics were added if desired. These bacterial cells were cultured at 37°C with 200 rpm for 6 h.

### Preparation of samples for nitrite-reprogrammed metabolome and GC-MS analysis.

Preparation of samples for nitrite-reprogrammed metabolome was performed as previously described (19). In brief, bacteria were cultured with or without nitrite at 37°C with 200 rpm for 6 h. Then, the bacteria were collected and washed three times with saline. The cells were suspended in saline and adjusted to OD_600_ nm at 1.0. Aliquot of 10 mL cells were collected and transferred to a 1.5 mL centrifuge tube. The cells were immediately quenched with −80°C precooled methanol (HPLC grade). Resuspension of the quenched cells was sonicated for 10 min (200 W total power with 35% output, 2 s pulse, 3 s pause over ice) when 10 μL 0.1 mg/mL ribitol (Sigma) was added as the internal standard. Supernatant was separated by centrifugation at 4°C and 12,000 × *g* for 10 min and placed in a 37°C vacuum centrifuge dryer (Labconco, USA) to evaporate the methanol. The dried samples were added to 80 μL of 20 mg/mL methoximation-pyridine hydrochloride (Sigma-Aldrich) at 37°C and 200 rpm for 3 h. Subsequently, 80 μL of N-methyl-N-trimethylsilyltrifluoroacetamide (MSTFA; Sigma-Aldrich) was added and the reaction was performed at 37°C and 200 rpm for 30 min. The GC-MS data were detected by Agilent 7890A GC equipped with an Agilent 5975C VL MSD detector (Agilent Technologies). The samples of 1 μL were injected into a 30 m × 250 μm i.d. × 0.25 μm DBS-MS column. Initial temperature of the GC oven was held at 85°C for 5 min followed by an increase to 270°C at a rate of 15°C/min and then held for 5 min. Helium was used as the carrier gas and flow was kept constant at 1 mL/min. The MS was operated in a range of 50–600 *m/z*. Each sample was analyzed in four biological repeats with two technical replicas.

### Analysis of metabolomic data.

Statistical analysis was performed as previously described (38). Briefly, Agilent Chrom Station software (Agilent Technologies, USA) was used to analyze mass fragmentation spectrum and identify compounds thorough matching data with the National Institute of Standards and Technology (NIST) library and NIST MS search 2.0 program. The data were normalized based on total amount of correction and standardized data containing metabolites, retention times, and peak areas, and prepared for further metabolomics analysis. Significant difference of the standardized data was calculated and selected (*P* value <0.05) by software IBM SPSS Statistics 19. Cluster analysis was carried out by R software (*R *× 64 3.6.1). Normalized area of differential metabolites was analyzed with Z-score. Principal-component analysis and S-plot analysis were performed by SIMCA-*P + *12.0 software (version 12; Umetrics, Umea, Sweden), and the metabolic pathway was done with MetaboAnalyst 4.0 enrichment. Interactive Pathways (iPath) analysis was conducted with iPath 3.0 (https://pathways.embl.de/). Figures were draw by GraphPad Prism 7.0 and Adobe Illustrator CS6.

### Detection of nitric oxide.

Method for NO content determination under the concentration gradient of nitrite was performed as previously described (19). In brief, bacteria were cultured in M9 medium with or without metabolites at 37°C with 200 rpm for 6 h. Cells were collected and washed three times with saline. The cells were suspended in saline and adjusted to OD_600_ nm at 1.0. Aliquot of 30 mL cells were collected and transferred to a 1.5 mL centrifuge tube. The cells were resuspended with 0.6 mL saline and disrupted by sonic oscillation for 7 min (200 W total power with 35% output, 2 s pulse, 3 s pause over ice). After centrifugation at 12,000 × *g* for 10 min at 4°C, supernatants were collected. Protein concentration of the supernatant was quantified by BCA protein concentration determination kit (Beyotime, P0009). Then, 500 μL of 4 mg/mL proteins were mixed with 400 μL buffer I at 37°C for 60 min. The reaction solution was added to 300 μL buffer II, vortexed, and mixed well for 30 s, and then sedimented at 25°C for 40 min. Supernatant was obtained by concentration at 1,600 × *g* for 10 min. Aliquot of 800 μL supernatant was mixed with 600 μL color developer and sedimented at 25°C for 10 min. Absorbance was measured at 550 nm with a cuvette with 0.5 cm optical path. The same volume of protein buffer and 0.1 mmol/L standard samples were used as a blank control and a reference for concentration calculation, respectively. NO concentration (μmoL/g) was calculated as follows: NO concentration = (Experiment group OD value – blank OD value)/(standard sample OD value – blank OD value) × standard sample concentration/protein concentration.

### Measurement of enzyme activity.

Bacteria were cultured in M9 medium with or without nitrite at 37°C with shaking at 200 rpm for 6 h. Cells were collected and immediately snap-frozen in liquid nitrogen and stored at −80°C. The cells were resuspended with 0.5 mL of 1× PBS and disrupted by sonic oscillation for 5 min (200 W total power with 35% output, 2 s pulse, and 3 s pause over ice). The protein concentration of the supernatant was quantified with a bicinchoninic acid (BCA) kit (Beyotime, P0009, China), and the final protein concentration for each sample was diluted to 3 mg/mL. Complex I activity was determined at 340 nm with a complex I activity assay kit (Solarbio BC 0515, China). Complex III activity was determined at 550 nm with a complex III activity assay kit (Solarbio BC 3240, China). SOD and CAT activity were measured at 450 nm and 405 nm, respectively, using an SOD and CAT assay kit from Nanjing Biomedical Research Institute of Nanjing University (A001-3-2 and A007-1-1, China). PDH, KGDH, SDH, and MDH activities were measured as described previously (19). In brief, a reaction mixture contained 0.15 mM 3-(4,5-dimethyl-2-thiazolyl)-2,5-diphenyl-2H-tetrazolium bromide (MTT), 2.5 mM MgCl_2_, 6.5 mM phenazine methosulfate (PMS), 0.2 mM thiamine PPi (TPP), and 80 mM sodium pyruvate/alpha-ketoglutaric acid potassium salt for PDH and KGDH activity measurement. Another reaction mixture contained 0.15 mM MTT, 2.5 mM MgCl_2_, 13 mM PMS, and 80 mM sodium succinate/sodium malate for SDH and MDH activity measurement. Then, the reaction mixtures were incubated at 37°C for 5 min for MDH, PDH, and KGDH, and 10 min for SDH and detected at 562 nm for colorimetric readings. Experiments were repeated in at least three independent biological replicates.

### Quantitative real-time PCR.

To investigate the effect of nitrite on gene expression levels, quantitative real-time PCR (qRT-PCR) was performed as previously described (39). In brief, after incubation in M9 with or without nitrite at 37°C with 200 rpm for 6 h, cells were collected and adjusted to 1.0 of OD_600_ nm. Total RNA was isolated from 1 mL cell samples by TRIzol reagent (Invitrogen Life Technologies). cDNA was obtained from 1 μg total RNA, and reverse transcription was performed according to a PrimeScript RT reagent kit with gDNA Eraser (Guangzhou IGE Biotechnology Ltd., China). The primers used for qRT-PCR are listed in Table 1, where the 16S rRNA gene served as an internal control. The qRT-PCR was performed in 384-well plates with the SYBR Green Premix Pro *Taq* HS qPCR Kit (Guangzhou IGE Biotechnology Ltd., China) at a total volume of 10 μL. The reaction mixtures were run on a LightCycler 480 system (Roche, Germany). The cycling parameter values were set as follows: 95°C for 30 s to activate the polymerase, 40 cycles of 95°C for 5 s, 58°C for 30 s. Fluorescence measurements were performed at 75°C for 1 s during each cycle. Cycling was terminated at 95°C with a calefactive velocity of 0.11°C s^−1^ to obtain a melting curve. Data were calculated as relative mRNA expression compared to without nitrite group with the endogenous reference 16S rRNA gene.

**TABLE 1:** Primers for qRT-PCR

Gene	Primer sequence (5′–3′)	Gene	Primer sequence (5′–3′)
16S rRNA-F	CAAAACTACTGAGCTAGAGTACG	PA0105F	CACGGTGGAAATCCTCTGG
16S rRNA-R	TAAGATCTCAAGGATCCCAACGGCT	PA0105R	CGGCTCGGAAGTGTCGTAGAT
PA2637F	GGGGTTTCGTCCCTGCTT	PA0106F	CCTTCGGTGCGGTCAGT
PA2637R	CGATCCGCCAAAGATAGACA	PA0106R	TCAGCGAACCCTCCCACA
PA2638F	CGATAGGCGAACGGGAAAC	PA1553F	TGCCGAGACCGAACGCTAC
PA2638R	GCACGACAGCCCGAAGTTAT	PA1553R	CACGGGTAGGCAGGCATCT
PA2639F	GGCGACCAAGGGTATCAACA	PA1554F	GAATGGGTCTCGGTGTCCT
PA2639R	CCACGTCAGCCATAACGAAA	PA1554R	CAGTTCGGCGTATTCCTTG
PA2640F	CCGACCGTTTCGTCCTCAG	PA5554F	CGTGGGCAAGACCGTCAACA
PA2640R	GACCGATGCCGAGTTGCTT	PA5554R	GGTAGATACCCAGCGAGGC
PA2641F	CGAGATGGAGCCGAACACC	PA5555F	ACGAACCCGACGCCAAGT
PA2641R	AGGGAAGGGCGGTTTGG	PA5555R	TTTCCGAGATTTCCTGGGTG
PA2642F	TGTCTGTCCCTCGGTCTCG	PA5556F	GGAGCAGGATTCCGTGGGT
PA2642R	GTCGTGCGGGTGGTTGGT	PA5556R	GCCGCTGTCTTTCTGGTTG
PA2643F	GCCCAGACCATCTCCTACGA	PA5558F	CCGTTGCGTTCTTCATCTTTG
PA2643R	AGTTCCTGCTCCGCTTCCG	PA5558R	CCTTGACGCTGTTCAGTTCCT
PA2644F	GCTTCGCAGCCTGGTGATG	PA5559F	TGACTGCTATCGCCGTTGC
PA2644R	CGGCTCTTCGGGGTATTGC	PA5559R	TCCAGGAACTTGCCACCC
PA2645F	TGGTTCTGTTCGTCTTCGTGG	PA5560F	CCGCTTCGGGTTACATCCA
PA2645R	CGCCTTGGCATCTACCGTG	PA5560R	CCCATTTCCTTGGCTTGCTC
PA2646F	GATGAGCCTGGAAGTGATGATG	PA4468F	CTTGCCGCCCTTGCCTTAC
PA2646R	GGTGTGGAAGCGGCGATA	PA4468R	TGCCGCAGCAGACTTTCCA
PA2647F	TGCTGCTGTCGTTCTCCCG	PA4366F	AGCCGCACATTTCCGCA
PA2647R	CGTTGTTGCGATTGCTGTAGTA	PA4366R	GGGCTCAGGCAGTTCCAGTA
PA2648F	GGCGTGCTCTCGGTCCTC	PA4613F	GAACGCCTTCCGTCCCG
PA2648R	TTGCCGTCATCGGAACTGT	PA4613R	GCGGATGAAGAAGGTCGGG
PA2649F	TGAACATCTCCCTGAGCGTC	PA4236F	ATGCGACCTTCAAGTGGGA
PA2649R	GCCCCAGTTGAACGGTGC	PA4236R	GGGTAGTCACCGTGGGGC
PA2691F	TGCCCTGATGACCTGCCA	PA2147F	GAAGGGCGACTATCCCGAAT
PA2691R	ATCTTCTTGCCCTCCTCGC	PA2147R	CAGCGGGTCGTTGGTGA
PA4538F	AACCTCACCCACATCTGGAAAC	PA5240F	GCCCTGTGCTGGTGGACT
PA4538R	GATACTCACTGCGTTGTTGTTCG	PA5240R	TTCGGCGGGGTATCCTG
PA1581F	TCAAACTCCCTGTCACCGCT	PA2616F	CCTGGACGAGGTGCTGGG
PA1581R	CACGCTTACCGCCTTCCA	PA2616R	GGTCGGTGTTCGGCTTGTG
PA1582F	TGTCATTTTCCTGCTGGGCT	PA0139F	GCTGCCTTCACCTTCAACTGC
PA1582R	GTCCACATACCGACCCACG	PA0139R	GGACGGAGCGAGGGTCTTCT
PA1583F	CGACCCGAACGACGATTG	PA1008F	CCGACTTCACCGCCCCC
PA1583R	CGAACGGACGCTGGTAGATG	PA1008R	TGGCAGACCGCTTCATCCT
PA1584F	TCAAGGAACAGGACGAGGGC	PA0407F	GATGCCTCCCGCTGGTTC
PA1584R	AGAAGGACGGGCAGGAGG	PA0407R	AACTGCGTGGCGAAGAACT

### Antibiotic bactericidal assay.

As previously described, antibiotic bactericidal assay was performed in M9 with or without metabolites or/and antibiotics and incubated at 37°C with 200 rpm (40). Six hours later, 100 μL of samples were 10-fold continuously diluted and 10 μL of each dilution was aliquoted on the 2% LB agar plates and cultured at 37°C for about 11 h, and CFU per mL was determined. The percent survival was determined by dividing the CFU obtained from the treated sample by the CFU obtained from the control.

### Determination of membrane potential.

The membrane potential (PMF) of AP-R_CLIN-EVO_ and AP-R_LAB-EVO_ with or without nitrite was measured as follows: bacteria were cultured with or without 20 mM nitrite and incubated at 37°C with 200 rpm for 6 h. Then, the cells were labeled by 3 mM DiOC_2_ (Sigma) in the dark at 30°C for 30 min. The samples were analyze using a BD FACSCalibur flow cytometer with an excitation wavelength of 488 nm and emission wavelength of 610 nm. Gates for bacterial populations were based on the control population by using forward versus side scatter and red versus green emission. The diverse ratios of red and green indicated fluorescence intensity values of the gated populations. Membrane potential was calculated according to the following: log(10^3/2^ × [red fluorescence/green fluorescence]). Three repetitions were performed.

### Reactive oxygen species (ROS) assays.

ROS assays were performed as follows: bacteria were cultured in M9 with or without metabolites or/and antibiotics at 37°C with 200 rpm for 6 h. A microplate reader was added, and 10^7^ CFU cells and 10 μM 2’,7’-dichlorfluorescin diacetate (Sigma) were incubated at 37°C for 30 min in the dark. The samples were analyzed by a Victor X5 multimode plate reader at excitation and emission wavelengths of 485 nm and 535 nm, respectively.

### Data availability.

All of the metabolomic raw data were deposited to MetaboLights (http://www.ebi.ac.uk/metabolights/) (41). The unique identifier is MTBLS4346.
